# The moderating role of social support in the relationship between death anxiety and resilience among dialysis patients

**DOI:** 10.1186/s12882-024-03533-x

**Published:** 2024-03-16

**Authors:** Zahra Shafiei Kisomi, Omid Taherkhani, Mina Mollaei, Hoda Esmaeily, Ghazaleh Shirkhanloo, Zahra Hosseinkhani, Mohammad Amerzadeh

**Affiliations:** 1 Social Determinants of Health Research Center, Qazvin University of Medical Sciences, Qazvin, Iran; 2 Student Research Committee, Qazvin University of Medical Sciences, Qazvin, Iran; 3 Metabolic Diseases Research Center, Research Institute for Prevention of Non-Communicable Diseases, Qazvin University of Medical Sciences, Qazvin, Iran; 4 Social Determinants of Health Research Center, Research Institute for Prevention of Non-Communicable Diseases, Qazvin University of Medical Sciences, Qazvin, Iran; 5 Department of Healthcare Management, School of Public Health, Qazvin University of Medical Sciences (QUMS), Qazvin, Iran

**Keywords:** Social support, Anxiety, Resilience, Dialysis

## Abstract

**Background:**

Chronic Kidney Disease (CKD) who receive social support can cope with the challenges. Therefore, this study determined the moderating role of social support in the relationship between death anxiety and resilience among dialysis patients in Qazvin City.

**Methods:**

This cross-sectional study used a descriptive-analytical approach on 347 dialysis patients in Qazvin City. The data collection tools included several questionnaires. The convenience sampling method was employed. The data were analyzed using SPSS software version 22 and mplus software version 7.2, employing descriptive statistics, such as mean and standard deviation for continuous variables and using counts and percentages for categorical/nominal variables. Regression analysis and tests were used to examine the relationships between variables. Structural Equation Modeling (SEM) analysis was employed to determine direct and indirect relationships between independent and dependent variables.

**Results:**

The prevalence of death anxiety was high (48.3%) among the patients. The mean resilience score was 62.59 ± 15.69, and the mean social support score was 52.23 ± 10.21. There was a significant association between resilience and social support (*P* < 0.001), as well as between resilience and death anxiety (*P* < 0.001). Furthermore, a significant relationship was observed between social support and death anxiety (*p* = 0.015). In the analysis of SEM, both the direct and indirect relationships between resilience and death anxiety were significant through the mediating variable of social support.

**Conclusion:**

This study demonstrates that there is a relationship between death anxiety and resilience, and social support significantly moderates the relationship between death anxiety and resilience.

## Background

Chronic kidney disease (CKD) is one of the most common chronic non-communicable diseases [1] and is among the top 20 leading causes of death worldwide [2]. In this disease, the kidneys gradually become damaged or cannot clean the blood like healthy kidneys. As a result, waste products and excess fluid accumulate in the body, leading to various health problems, including heart disease and high blood pressure [3]. Currently, 2 to 3% of the global population is facing the challenge of chronic kidney failure. Approximately 2.6 million people worldwide are living with alternative methods, such as hemodialysis and kidney transplantation, and it is predicted that this number will reach 5.5 million by 2030 [4]. Hemodialysis is one of the methods used to control and treat CKD. Although dialysis and other treatment methods have, to some extent, reduced disease symptoms, increased lifespan, and improved the quality of life of patients, their family and social aspects are affected by the disease and treatment complications, and many patients face disability and mental disorders [5, 6]. Several studies indicate a high prevalence of psychosocial disorders in dialysis patients. Depression, interpersonal sensitivity, paranoid thoughts, somatic complaints, obsessive-compulsive thoughts, anxiety, psychosis, aggression, and morbid fear are among the common psychiatric disorders in dialysis patients [7].

One of the most common psychological disorders that leads to a reduction in the quality of life among dialysis patients is anxiety. The prevalence of anxiety in hemodialysis patients within the country ranges from 20 to 60%. The duration of treatment and deficiencies in the support system of hemodialysis patients result in their incapacity to cope with stressful situations and an increase in anxiety. Types of anxiety have been categorized based on their origins, and death anxiety is one of the most important categories. It is defined as the fear of one’s impending death, the fear of losing significant people in one’s life, and adverse emotional reactions triggered by the individual’s anticipation of nonexistence [8]. Contemplating death can be terrifying, and most people prefer not to think about it. Patients undergoing hemodialysis treatment also face the fear of death due to the stressful conditions of their lives [9]. Several factors can contribute to reducing anxiety associated with chronic illness, and resilience is one of them [10].

Resilience, as a dynamic process, can lead to positive adaptation in facing unpleasant experiences in life for individuals with chronic illnesses, enabling them to manage anxiety-provoking and stressful situations better. Resilience can enhance individuals’ self-esteem, emotional stability, and personal strength. Individuals with high levels of resilience have a remarkable ability to cope with the challenges related to their illness. As a result, adaptation to chronic diseases occurs more rapidly in individuals who possess facilitators, such as resilience, problem-solving skills, hope, spirituality, etc. Resilience is expressed as an essential form of self-protection in individuals with CKD who experience various sources of stress [11]. By improving resilience, these patients can withstand stress factors, anxiety, and psychological problems [12]. One way to enhance resilience is by having social support for patients [11].

Social support is physical and psychological support provided to the patient, typically by family, friends, coworkers, spiritual counselors, healthcare professionals, and community members. It can lead to improved health and an enhanced quality of life for dialysis patients [13]. Social support can reduce negative emotions and physiological responses, improve physical health [14], and facilitate long-term treatment, success, and patient adaptation. Social support is critical in the treatment process of CKD patients and helps better control stress [15]. Due to the extensive impacts of the disease on all aspects of life, these patients belong to the most vulnerable population in society [16] and have a significant need for social support from family, friends, and others in their lives. Furthermore, due to lifelong dialysis treatment, these individuals experience impairments in daily functioning and social activities [17, 18]. As a result, their interactions with others become more limited daily, and their need for social support increases [19–21].

Evidence suggests a strong relationship between social support and increased resilience against illness [22]. The findings of Newton et al. indicate a significant association between higher resilience and better social support [23]. Resilience and social support can mutually influence each other, and increased psychological resilience may lead to more positive interactions [11]. Therefore, besides caring for physical health, promoting and developing the mental health of patients with chronic illnesses is highly important [24]. Higher resilience is associated with greater acceptance of the illness, better adherence to treatment regimens, and improved outcomes for patients with CKD [25, 26]. Besides, according to the buffering effect in the social support theory, individuals who receive social support are better able to cope with the conditions of their illness and better manage the stressful aspects of their disease [27].

Additionally, there is a significant relationship between social support and the level of resilience in chronic patients, in such a way that social support positively influences mental health and directly reduces stress. In contrast, the availability of social support resources minimizes stress-inducing events in life, thus reducing the risk of physical and mental illnesses [11, 22]. However, the study’s results by Vaghase et al. present conflicting results regarding these two components. In this study, no significant difference in scores on the resilience scale was reported between two groups with different levels of received social support because the researchers believe that despite experiencing traumatic events, many individuals continue to maintain hope and flexibility [28].

According to the available evidence, social support plays a crucial role in achieving two important goals: improving the quality of life and overcoming chronic stress in patients with end-stage renal disease (ESRD) [29]. It can enhance patients’ resilience and help them adapt independently [30, 31]. Studies have shown that social support is a significant factor in psychological adaptation and resilience against chronic illnesses [32]. Individuals with social support can cope with stressful events in life, reducing the risk of various physical and mental illnesses following a chronic disease [11]. However, the moderating role of social support in death anxiety and resilience among dialysis patients has been less studied worldwide, and its impact on reducing death anxiety and increasing resilience in patients is largely unknown. Moreover, the few available studies in Iran have only addressed this disease’s overall impact on patients’ quality of life [22, 33].

As supporters of patients dealing with chronic diseases, especially those undergoing dialysis, nurses can contribute to forming an adequate social support system for patients and play an influential role in enhancing their resilience and recovery. Additionally, dialysis nurses have an essential role in integrating medical and psychiatric aspects into patient care, enabling them to plan more effective care for the problems faced by these patients [11]. Therefore, the present study aimed to investigate the moderating role of social support in the relationship between death anxiety and resilience among dialysis patients, referring to the educational center of Bu-Ali Sina in Qazvin City.

## Methods

### Study design

This is a cross-sectional, descriptive-analytical study examining the moderating role of social support in the relationship between death anxiety and resilience among dialysis patients referring to the educational center of Bu-Ali Sina in Qazvin City during 2022–2023.

### Sample size

All individuals who met the inclusion criteria were invited to participate in the study after comprehensively explaining the study’s objectives and methodology. Written informed consent was obtained from each participant. The inclusion criteria for this study included literacy in reading and writing, proficiency in the Persian language, willingness to participate in the study, and confirmation of CKD by a specialist. The exclusion criteria included incomplete questionnaire completion, inability to communicate in Persian, and withdrawal from completing the questionnaires at any stage of the response process.

In this study, the researcher completed the questionnaires through interviews with the participants. The sampling method used was convenience sampling. The data were collected.

Given the utilization of Structural Equation Modeling (SEM) analysis and the number of questionnaire items and variables under investigation, a minimum of 5 participants per item was considered. Considering participant attrition and sample size requirements, a minimum sample size of 347 individuals was determined.

### Data collection

To gather data, a four-part questionnaire was employed:

- Demographic Checklist: This checklist encompassed age, gender, marital status, occupation, economic status, educational level, duration of disease diagnosis, history of kidney transplantation, number of dialysis sessions per week, number of siblings, place of residence, and insurance status.
- Resilience Scale: This scale, designed by Connor-Davidson (CD-RISC), consisted of 25 items rated on a 5-point Likert scale ranging from 0 to 4 (completely untrue − 0, rarely true − 1, sometimes true − 2, often true − 3, and true nearly all the time − 4) [34]. It assessed the construct of resilience. The minimum possible score on this scale is zero, and the maximum score is 100. A higher score indicates greater resilience in the individual. The psychometric properties of this scale have been investigated in six population groups, including the general population, primary care attendees, psychiatric outpatients, individuals with generalized anxiety disorder, and two groups of post-traumatic stress disorder patients. The developers of this scale assert that it can effectively differentiate between resilient and non-resilient individuals in both clinical and non-clinical groups and can be used in research and clinical settings [35]. Preliminary studies on this scale’s psychometric properties, reliability, and validity have been conducted and confirmed [36]. The reliability and validity of the Persian version of the resilience scale have also been examined and confirmed [37]. In studies conducted on hemodialysis patients, reliability was assessed using Cronbach’s alpha, and Cronbach’s alpha coefficients for each resilience subscale ranged from 0.73 to 0.91 [38–40].
- Death Anxiety Scale: The Death Anxiety Scale by Templer consists of 15 items. It was initially introduced by Templer in 1970. Each item is answered with either “yes” or “no,” with a score of 1 assigned to “yes” responses and a score of 0 assigned to “no” responses. A “yes” response indicates the presence of death anxiety in the individual, while a “no” response indicates the absence of death anxiety. Scores on this instrument range from 0 to 15, with scores of eight or higher indicating high levels of death anxiety, and scores below seven indicating low levels of death anxiety. Templer reported a test-retest reliability coefficient of 0.83 for this scale [41]. In Iran, Rajabi et al. investigated the validity of this scale and reported a split-half reliability coefficient of 0.62 and a Cronbach’s alpha coefficient of 0.73. They also examined the criterion validity of the death anxiety scale using two other instruments, the death concern scale and the manifest anxiety scale, which yielded correlation coefficients of 0.40 and 0.43, respectively [42]. The validity and reliability of this scale have been examined and confirmed in previous studies as well [43–45]. In a study conducted on hemodialysis patients, reliability was assessed using Cronbach’s alpha, and the Cronbach’s alpha coefficients for each subscale of death anxiety were 0.835 [46].
- Social Support Scale: The Social Support Assessment (SS-A) questionnaire was developed in 1986 to measure an individual’s perceived social support. This scale is based on Cobb’s definition of social support, which refers to the extent of receiving love, assistance, and attention from family members, friends, and other individuals. The questionnaire consists of 23 items rated on a 5-point Likert scale, ranging from “strongly disagree” to “strongly agree.” It includes three subscales: family support, friend support, and other support [47]. The scoring is as follows: very low social support: below 41, low social support: 41–59, moderate social support: 59–77, low social support: 77–95, very low social support: 95–115. In the study conducted by Saadat et al., the validity of the questionnaire was confirmed by experts in the field, and the reliability of the questionnaire was assessed using Cronbach’s alpha, resulting in a coefficient higher than 0.70 [48].

### Data analysis

The data were analyzed using SPSS software version 22 and mplus software version 7.2, employing descriptive statistics, such as mean and standard deviation for continuous variables like age, and using counts and percentages for categorical/nominal variables like gender. Regression analysis and tests were used to examine the relationships between variables. SEM analysis determined direct and indirect relationships between independent and dependent variables. The maximum likelihood (ML) method is used in the SEM analysis model, which includes two parts: measurement model and structural model, and the estimation of the model is by determining the size of the regression between the latent and observed variables. To conduct the SEM model, the necessary assumptions, including the normality of the data, the adequacy of the sample size, the type of missing pattern in the data, and the existence of the minimum number of observational variables (3–5 variables) for each latent variable were checked.

## Results

The mean age and standard deviation of the participants in the study were 56.75 (± 15.21) years. The gender of the participants was 63.37% male and 36.63% female. The majority of participants were married (81.1%). Most participants had a high school diploma or a lower level of education (83.1%). The economic status of the participants was average primarily or low (93.1%). The demographic and clinical characteristics of the patients are presented in Table 1. In the analysis of the relationship between demographic and clinical variables with death anxiety in the univariate analysis, significant associations were observed between the number of siblings (*P* = 0.010), duration of diagnosis (*P* = 0.031), number of dialysis sessions (*P* = 0.026), education level (*P* = 0.001), place of residence (*P* = 0.001), insurance status (*P* = 0.002), and economic status (*P* < 0.001) of the participants. In the multivariate analysis, after adjusting for confounding variables, significant associations were found only for the variables of number of siblings (*P* = 0.006), education level (*P* = 0.014), history of kidney transplantation (*P* = 0.040), duration of diagnosis (*P* = 0.003), number of dialysis sessions (*P* = 0.020), place of residence (*P* = 0.024), and economic status (*P* = 0.003) of the participants (Table 1).

**Table 1:** The demographic variables and clinical characteristics of the patients and their association with death anxiety

Variables	Mean(SD) N(%)		Crud estimate			Adjusted estimates		
			Coefficients	SE	*P* value	Coefficients	SE	*P* value
Age	56.75(15.21)		0.006	0.003	0.068	-	-	-
Number of siblings	3.7(1.90)		-0.07	0.03	0.010	-0.07	0.03	0.006
Duration of diagnosis	51.68(51.00)		-0.002	0.001	0.031	-0.003	0.001	0.003
Number of dialysis sessions	4(2.93)		0.42	0.19	0.026	0.42	0.18	0.020
Gender	Female	127(36.3)	-0.13	0.11	0.252	-	-	-
	Male	223(63.7)						
Occupation	Yes	164(46.9)	-0.04	0.11	0.685	-	-	-
	No	186(53.1)						
Education level	Literate	67(19.1)	-0.13	0.04	0.001	-0.10	0.04	0.014
	Primary school	73(20.9)						
	Junior school	70(20.0)						
	High school	88(25.1)						
	Academic	52(14.9)						
Marital status	Single	49(14.0)	0.21	0.12	0.095	-	-	-
	Married	284(81.1)						
	Divorce	17(4.9)						
History of kidney transplantation	Yes	69(19.7)	-0.14	0.13	0.279	-0.29	0.14	0.040
	No	281(80.3)						
Economic status	Poor	116(33.1)	-0.38	0.09	< 0.001	-0.27	0.09	0.003
	Medium	210(60.0)						
	Rich	24(6.9)						
Place of residence	City	270(77.1)	0.43	0.12	0.001	0.30	0.13	0.024
	Village	80(22.9)						
Insurance status	Yes	303(86.6)	0.47	0.15	0.002	-	-	-
	No	47(13.4)						

The level of death anxiety was high in 51.7% (181 individuals) of the patients and low in 48.3% (169 individuals) of the patients. The average resilience score was 59.62 (± 15.69), indicating moderate resilience. The average social support score was 52.23(± 10.21), which was reported as 31.77% after standardization, indicating a relatively low level of social support.

**Table 2:** The Effects (standard errors) of variables in SEM model

Variables	Total effect			Direct effect			Indirect effect		
	**Coefficients**	**SE**	***P*** **value**	**Coefficients**	**SE**	***P*** **value**	**Coefficients**	**SE**	***P*** **value**
	**0.313**	**0.048**	*P* < 0.001	**0.394**	**0.057**	*P* < 0.001	**-0.081**	**0.034**	*P* = 0.018
Goodness of fit									
χ2 (df)	**398.108** [10]								
RMSEA	**0.084(0.033, 0.143)**								
CFI/ TLI	**0.981/0.936**								
SRMR	**0.035**								

**Fig. 1:**
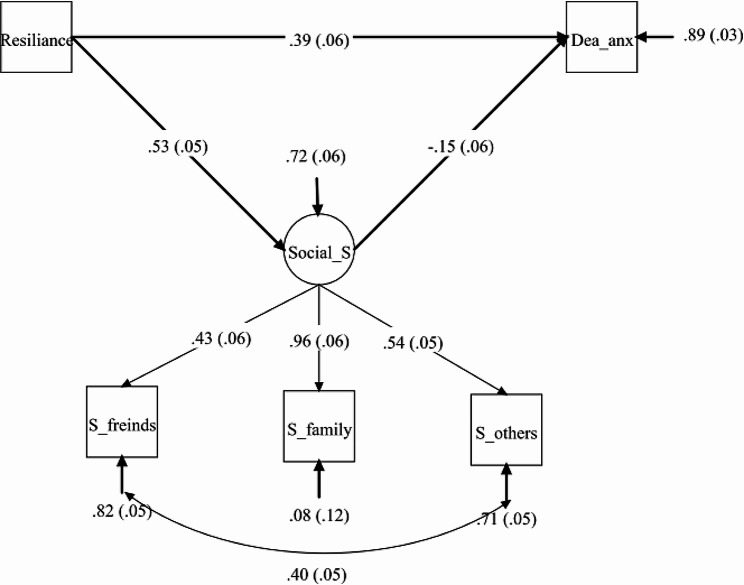
SEM with death anxiety as outcome, the resilience as predictor and the social support as mediator. All paths are significant at *P* < 0.05 and standardized coefficients are shown

In the SEM analysis to determine the direct and indirect relationship between resilience and death anxiety, using social support as a mediating variable, all paths were found to be significant. The causal model based on the extracted data indicators fits well (SRMR = 0.035, CFI = 0.936, GFI = 0.981, χ2(df) = 398.108 [10]). The direct relationship between resilience and death anxiety was significant. Additionally, the indirect relationship between the two variables through the mediating variable of social support was also significant, indicating that social support plays a significant role in the relationship between resilience and death anxiety (Table 2; Fig. 1).

**Table 3:** The estimate (SD) in the association between variables in SEM analysis

Variables	Un-standardized			standardized		
	Coefficients	SE	*P* value	Coefficients	SE	*P* value
Resilience→Social support	0.23	0.05	*P* < 0.001	0.53	0.05	*P* < 0.001
Social support→Death anxiety	-0.35	0.15	*P* = 0.022	-0.15	0.06	*P* = 0.015
Resilience→Death anxiety	0.39	0.06	*P* < 0.001	0.04	0.06	*P* < 0.001

As shown in Table 3, there is a significant relationship between resilience and social support (*P* < 0.001), as well as between resilience and death anxiety (*P* < 0.001). The table indicates that 53% of the variance in social support is explained by resilience. In comparison, 15% of the variance in death anxiety is explained by social support, albeit with an inverse effect. Furthermore, a significant relationship was observed between social support and death anxiety (*P* = 0.015). However, in the relationship between resilience and death anxiety, although statistically significant, only 4% of the variance is explained.

## Discussion

The study found a significant relationship between resilience and death anxiety in hemodialysis patients, and social support significantly moderated this relationship. Furthermore, the findings of the mediation model demonstrated that social support acts as a third variable or mediator and plays a significant role in the relationship between resilience and death anxiety.

Previous studies have reported a negative association between high levels of social support and the occurrence of illness and death [49, 50]. Studies conducted in Pakistan among patients with chronic diseases revealed that social support is a crucial factor for patients with chronic diseases to cope with life-threatening conditions [49–51]. Social support has a vital role in the rapid recovery of hemodialysis patients. There is a negative relationship between death anxiety and social support, as social support helps reduce the level of death anxiety and increases the period of recovery in chronic patients [51].

Alsharif et al. [52] and Pan et al. [53] demonstrated that social support plays a robust mediating role in reducing symptoms of depression and sleep disorders and improving the quality of life in hemodialysis patients. Social support is one of the most effective methods for long-term treatment and adaptation to the disease, usually provided by family, friends, colleagues, spiritual counselors, mental health professionals, and community members [15]. Lilliepakki et al. also reported that patients with high levels of anxiety and depression had less support from significant others, family, and friends [54]. Cha’s study on the self-care model of hemodialysis patients showed that support from family, friends, colleagues, and healthcare professionals significantly impacted self-care behaviors [55]. Social support is a valuable coping technique and a source of effective adaptation that paves the way for love, affection, expression of existence, self-awareness, and a sense of belonging. Even if it cannot overcome stressful situations in certain circumstances, it enables individuals to be more optimistic by reducing anxiety and increasing self-esteem. It may also help individuals cope with challenging situations and create new solutions, making them more satisfied with life [14, 56].

In the present study, social support significantly moderated the relationship between death anxiety and resilience. The reciprocal effect of death anxiety and social support was meaningfully associated with resilience. If such a moderator could be examined, it would help inappropriate treatment for coping with the fear of death [57]. Previous studies have shown a negative relationship between spiritual well-being and death anxiety. A resilient individual can think positively, easily control negative emotions, and have self-confidence [17, 58].

Furthermore, Zhang et al. demonstrated a significant association between resilience and quality of life in breast cancer patients, with social support mediating between resilience and quality of life in these patients [59]. Resilience may serve as a mechanism for patients to cope with the challenges they experience [60, 61]. Resilience is a fundamental factor in managing chronic diseases [62]. Additionally, low resilience is associated with higher risks of depression and fewer health-promoting behaviors among hemodialysis patients [60, 63]. Therefore, nurses have a significant responsibility to enhance the psychological resilience of patients to cope with the stressors associated with chronic illness and prevent mental disorders.

Our findings are inconsistent with the study conducted by Ong et al., which examined the moderating and mediating effects of perceived social support on the relationship between resilience and caregiver burden in the care of the elderly [64]. In this study, researchers showed that the social support variable had no moderating effect and could not influence the direction of the relationship between resilience and caregiver burden. Additionally, some studies question the positive effects of social support on parenting [65–68]. The difference in these results may be due to variations in the studied population, culture, quality of care, received social support, mental and psychological status, and economic conditions in the research settings of these two studies.

According to the significant correlation between educational level and death anxiety among hemodialysis patients in this study, consistent with previous findings, it was found that the death anxiety of patients decreases with an increase in their educational level [69–71]. Higher levels of awareness in individuals with higher educational levels, practical problem-solving skills, more accessible access to healthcare information, better management of their illness, and better socio-economic conditions may have contributed to increased psychological resilience. However, our study results did not align with other studies [51, 72].

Another factor that had a significant correlation with death anxiety in this study was the economic level. Similarly, previous studies also indicated that hemodialysis patients with lower income levels had higher death anxiety [73]. A higher income level is an important determinant of quality of life and a protective factor that affects death anxiety. This outcome may be due to patients with higher economic and educational status having more access to informational resources related to death, which subsequently reduces death anxiety in these individuals. Furthermore, our study findings indicated no statistically significant relationship between death anxiety and gender. This is in contrast to the study conducted by Shafiei et al. and Fathi et al., which showed that fear of death is more prevalent among men, leading to higher death anxiety, possibly due to the suppression of emotions such as fear in men [46, 72].

In this study, the place of residence had a significant correlation with death anxiety. It was observed that patients residing in rural areas had lower levels of death anxiety, which is consistent with the study conducted by Gholami et al. [69]. Additionally, in the study by Fathi et al., it was found that living in rural areas was associated with lower levels of death anxiety. However, this difference was not statistically significant [70]. It seems that the different family structure in rural areas, which is often more traditional and involves more significant contact with extended family members, results in a higher level of support and care for meeting their needs, leading to lower levels of death anxiety [69]. Furthermore, the presence of greater tranquility in the rural environment compared to the stressed conditions and issues present in urban areas is an undeniable factor.

### Implications for clinical practice

Social support has a positive relationship with the resilience of hemodialysis patients. Resilience is negatively associated with death anxiety. Resilience increases with increased social support. Therefore, if we can enhance the psychosocial support systems for patients, they will have higher psychological resilience and experience less death anxiety. Nurses have an essential responsibility to assist these patients in dealing with the challenges, improving their social support systems, and enhancing their psychological well-being. Nurses can consider the significant role of social support in increasing the resilience of hemodialysis patients when developing educational programs for patients and their families. Additionally, they can provide supportive resources and counseling services and establish support groups to develop social support skills for families if necessary.

### Limitations

Firstly, the findings of our study cannot be generalized to hemodialysis patients in other regions since the participants were selected from a single center. Secondly, the self-report nature of the questionnaires used can introduce reporting biases. Although we adjusted for demographic factors in our analysis, there may still be confounding factors remaining, such as daily mood fluctuations and comorbidities.

## Conclusion

The findings of this study demonstrated a relationship between death anxiety and resilience, and social support significantly moderates the relationship between death anxiety and resilience. Resilience plays a crucial role in managing chronic illnesses and improving health promotion behaviors in patients. Increasing patients’ resilience in coping with stressors related to chronic illness and preventing mental disorders while reducing death anxiety has a significant impact. Besides, social support can enhance resilience in hemodialysis patients. Therefore, considering the moderating role of social support, practical steps can be taken towards overcoming stressors, coping with the fear of death, and reducing death anxiety in these patients.

## Data Availability

The datasets used and/or analyzed during the current study available from the corresponding author on reasonable request. The entire dataset is in Farsi language. The Data can be available in English language for the readers and make available from the corresponding author on reasonable request.
